# When psychiatry does not learn: Epistemic humility, ignorance culture, exclusion culture and diagnostic weaponisation

**DOI:** 10.1371/journal.pmen.0000741

**Published:** 2026-10-05

**Authors:** Laurence Cobbaert, Rosiel Elwyn, James Downs, Sandi James, Matthew Jackman

**Affiliations:** 1 Independent Researcher, Sydney, New South Wales, Australia; 2 National PTSD Research Centre, Thompson Institute, University of the Sunshine Coast, Birtinya, Queensland, Australia; 3 Independent Researcher, Cardiff, United Kingdom; 4 University of Melbourne, Melbourne, Victoria, Australia; 5 University of Sydney, Sydney, New South Wales, Australia; PLOS: Public Library of Science, UNITED KINGDOM OF GREAT BRITAIN AND NORTHERN IRELAND

## Abstract

Psychiatry combines clinical expertise with substantial institutional authority over diagnosis, treatment access, coercion, clinical records and the credibility assigned to people receiving care. This Essay examines how that authority can impede learning from recurrent warnings across professional misconduct, coercive care, diagnostic practices and iatrogenic harm. We propose institutional non-learning as a cross-cutting problem: professional interpretation can shape whose testimony is believed, which harms are measured, how events are recorded and whether reform is evaluated. Drawing on epistemic injustice, psychiatric survivor research, treatment-safety research, publication and outcome-reporting bias, and empirical studies of diagnostic bias, we develop a framework for epistemic reciprocity and accountability. It distinguishes occurrence from prevalence, recognises that treatment risk varies across populations and contexts, separates benefit from safety, applies reflexive scrutiny across clinical, academic and lived experience expertise, and extends representativeness to both research participants and the published evidence base. We use ignorance culture, exclusion culture, defensiveness and diagnostic weaponisation to describe recurring institutional functions. A responsibility culture requires independent scrutiny, prospective harm surveillance, lived experience governance, remedy and redress, and publicly evaluable evidence that practice has changed and safety has improved.

## 1. Introduction: Institutional non-learning

Psychiatry is not monolithic. Practices vary across jurisdictions, services and clinicians. Across these settings, however, psychiatric institutions exercise consequential interpretive authority over diagnosis, access to treatment, involuntary care, clinical documentation, risk assessment and the credibility assigned to people receiving care. When concerns arise, the relevant question extends beyond whether an individual clinician acted improperly. It also concerns whether institutions can recognise, investigate and learn from harms in which their own concepts, practices or power may be implicated [1–4].

Epistemic injustice provides a framework for this problem. Anderson [1] locates epistemic justice in social institutions; Carel and Kidd [2] analyse knowledge asymmetries in healthcare; and Bueter [5] argues that excluding patients from psychiatric classification produces preventable epistemic losses. Related scholarship examines testimonial injustice, hermeneutical injustice and epistemic silencing [3,6–9]. Building on this literature, we use institutional non-learning to describe how warning signals can be attenuated when the same systems define legitimate knowledge, classify the person reporting harm, determine what is recorded and decide whether reform is needed.

Psychiatry also has traditions of self-critique, supervision, attention to countertransference, philosophy of psychiatry and professional disagreement. These practices are valuable, but internal reflection does not itself demonstrate institutional learning. Learning becomes visible when concerns are detected, reforms are implemented, outcomes are measured and recurrence is reduced. Psychiatric survivor scholarship developed alongside, and sometimes against, professional accounts, generating knowledge from experiences of diagnosis, treatment, coercion and service use that conventional psychiatric research often poorly represented [10–14].

Throughout this Essay, ignorance culture, exclusion culture, defensiveness and diagnostic weaponisation are functional concepts for tracing how warning signals are handled. Their counterpoint is epistemic humility: recognising the limits of professional knowledge, remaining open to correction and calibrating claims to the evidence available [2,6].

## 2. Evidence, situated expertise and the visibility of harm

### 2.1. Evidence and the assessment of harm

Clarity about evidence begins by separating its tasks: identifying occurrence, experience and mechanism; estimating prevalence; and estimating causal treatment effects. Qualitative research can rigorously investigate experiences, meanings, contexts and processes, but does not by itself estimate population prevalence. Epidemiological studies can estimate frequency with appropriate sampling and ascertainment. Randomised trials can estimate causal effects under defined conditions; applicability elsewhere requires attention to participants, interventions, outcomes and settings [15]. Evidence-based practice also integrates research with clinical expertise and individual circumstances [16]. As Rowland argues, treatment outcome evidence alone does not establish a proposed mechanism of change or its exclusivity to one therapy [17].

Quantification is especially difficult when a phenomenon is not routinely sought. Jonsson et al. [18] found that 79% of 132 randomised trials of psychological interventions gave no indication that harms were monitored; only 3% described both adverse events and collection methods. Later reviews identify inconsistent definitions, terminology and assessment [19–23]. The Positive and Negative Experiences of Psychotherapy scale provides a patient-rated monitoring approach with preliminary psychometric support [24]. Limited safety evidence can consequently constrain guidelines and informed consent [25–27].

Negative experiences are not confined to isolated reports, although estimates depend on what is counted. Vybíral et al. [22] summarise estimates of 7% to 96% for at least one negative experience and 2% to 14% for reliable deterioration across different studies. These are not interchangeable outcomes. An adverse event occurs during treatment without necessarily being caused by it; iatrogenic harm requires an attribution to care. Negative experiences, deterioration and serious adverse events also differ in severity and ascertainment [21,22,24]. Combining them into one prevalence estimate would obscure clinically important differences.

Heterogeneity also concerns populations and contexts. Vybíral et al. [22] discuss identity disaffirmation, pathologising responses and value imposition affecting minoritised groups. Autistic people and parents describe neurotype-related misattunement and trauma in child and adolescent eating disorder care [28]. Lived experience-led synthesis identifies sensory, relational and intersectional pathways that conventional outcome frameworks may miss [29]. A sample with few or no members of a population cannot directly provide precise safety estimates for that population; extrapolation requires explicit justification, particularly when relevant outcomes are unmeasured [15,30]. This limitation does not establish either safety or harm in every unrepresented group.

Qualitative synthesis illustrates why outcome selection matters. Vybíral et al. [31] identified 21 meta-categories across 51 studies of clients’ negative psychotherapy experiences. Frequencies across studies are not patient prevalence estimates. Rather, this diversity helps identify what surveillance needs to measure, including processes and consequences that standard symptom scales may overlook.

### 2.2. Situated expertise and reciprocal scrutiny

Evidence also differs by standpoint. Lived experience can be treated as uniquely subjective while professional knowledge is presumed comparatively neutral [9,32]. Professional reasoning, however, is not a bias-free comparator. In Mendel et al.’s experimental diagnostic task, confirmatory information searching was associated with psychiatrists retaining an incorrect preliminary diagnosis [33]. Munder et al.’s meta-meta-analysis found an association between researcher allegiance and psychotherapy outcomes [34]; this does not, by itself, establish deliberate distortion or explain every allegiance effect. Blay et al. argue for scrutiny of ideological competing interests in psychiatric research [35]. These sources support reciprocal reflexivity without equating different kinds of expertise.

Diagnostic and therapeutic frameworks also embody conceptual and normative commitments. Historical and critical accounts examine how professional ideas shape diagnostic and treatment practices [36–38]. Hommel and Beste advocate an ideology-free mechanistic health psychology [39], whereas Ballesteros analyses how psychiatric standardisation can privilege particular forms of knowledge [40]. Indigenous scholarship identifies cultural and political assumptions within cognitive behavioural therapy [41]. Wainberg et al.’s strategic vision for the future Diagnostic and Statistical Manual of Mental Disorders emphasises socioeconomic, cultural, environmental and intersectional context [42]. Onstad analyses how therapeutic frameworks can constrain patients’ interpretive agency [43]. Their common implication is that professional premises warrant explicit examination rather than treating institutional dominance as evidence of neutrality.

Expertise is also shaped by what remains visible. Accounts of harm describe withdrawal from services after coercion, invalidation or poor treatment fit [44–47]. When feedback depends on continued engagement, such withdrawal may remove important perspectives from clinical learning. Research has analogous selection problems: harms may be inadequately assessed [18–21,24–27], while some null or unfavourable findings remain unpublished or selectively reported [48–52]. The implication is not that clinical experience lacks value, but that it can be informative and selectively constituted. Divergent accounts require examination of what each standpoint can access and how institutional power makes some interpretations authoritative.

Lived experience is increasingly recognised as expertise. The New South Wales Ministry of Health identifies a distinct lived experience workforce discipline [53], and The Lancet Psychiatry has introduced reporting expectations for lived experience involvement [54]. Banfield and Palmer argue for embedding this expertise throughout research [55]. In a structured collective reflection, Downs et al. describe the need for experiential knowledge to influence interpretation and governance [56]. Kious et al. question whether epistemic justice adds to good clinical practice and caution against indiscriminate acceptance of testimony [57]. Epistemic reciprocity does not require such acceptance: it requires matching expertise to the question and scrutinising professional interpretations as well as experiential accounts. Our focus extends to institutional arrangements that determine whose account can change practice [1,4,9].

### 2.3. Representativeness and the published record

Representativeness is relative to the population and question. Among 125 American Journal of Psychiatry articles, Pedersen et al. found that 43% reported race or ethnicity and 25% acknowledged limited representation as a limitation [58]. In 25 US and Canadian perinatal mental health studies conducted during the COVID-19 pandemic, Goyal et al. found that 76.5% of participants were White [59]. Egbert et al.’s eating disorder review found that fewer than half of sampled articles reported race or ethnicity; approximately 70% of participants in reporting studies were White [60]. Conversely, Dima et al. found that research participants’ ethnicity broadly reflected a London mental health Trust's eligible clinical population [61]. Large samples alone do not resolve underrepresentation, unmeasured outcomes or mismatched treatment contexts [15,30].

A separate question is whether publications represent the research conducted. Turner et al. compared 74 antidepressant trials with US Food and Drug Administration reviews: 94% appeared positive in the published literature versus 51% in regulatory assessments, and the published effect size was 32% larger overall [62]. Among 55 US National Institutes of Health-funded trials of psychological treatments for depression, Driessen et al. found that 23.6% were unpublished; including available unpublished results reduced the estimated effect by 25% [50]. Bowcut et al. identified outcome-related publication differences and protocol-publication discrepancies in schizophrenia and bipolar disorder trials funded by one nonprofit organisation [51]. These cohorts demonstrate specific distortions, not a uniform magnitude of bias across psychiatry.

Spencer et al. provide an informative contrast: all ten identified US National Institutes of Health grants for adult obsessive-compulsive disorder psychological-treatment trials resulted in publications [63]. That finding addresses non-publication in this grant cohort, not every form of selective reporting. Broader analyses identify mechanisms and remedies for reporting bias [48,49,52]. Completeness must therefore be investigated rather than presumed present or absent throughout a field.

Evidence should remain bounded by the questions, populations, outcomes and inferential assumptions it can support. This applies to claims of benefit and harm and to clinical, academic and lived experience expertise. Table 1 summarises these distinctions.

**Table 1:** Matching evidentiary claims to appropriate evidence.

Question	Best-suited evidence	What it cannot establish by itself	Interpretive risk
Does the phenomenon occur, and what is it like?	Qualitative interviews, narratives, case analyses and mixed-method research	Population prevalence	Conflating lack of a prevalence estimate with lack of evidence of occurrence
How common is it?	Representative surveys, registries, active surveillance, epidemiological studies, harmonised measures and subgroup estimates	A single generalisable rate when definitions, populations, exposures, instruments or ascertainment differ	Pooling non-equivalent constructs, populations or ascertainment methods
For whom and under what conditions is risk concentrated?	Stratified surveillance, adequately powered subgroup analyses, intersectional qualitative and mixed-method research, and deliberate inclusion of exposed communities	A universal risk estimate for groups, exposures or settings that were not represented	Applying an aggregate estimate uniformly across heterogeneous groups
Does an intervention cause an average benefit?	Well-designed comparative and randomised studies in defined populations	Universal effectiveness or safety across unrepresented populations	Extrapolating without justification beyond the studied population
Is an intervention safe?	Prospective harm definitions, active surveillance, long follow-up, patient-reported harms and triangulated data	Absence of harm from efficacy alone; causality from temporal association alone	Inferring safety from efficacy
Does the published literature represent the research conducted?	Trial registries, regulatory datasets, funder cohorts, protocols, unpublished study reports and protocol-publication comparisons	Population generalisability or absence of bias in unexamined subfields	Assuming the published record is complete
Whose expertise is relevant to the claim?	Lived experience, clinical, research and community knowledge matched to the question, with positionality and relevant blind spots made explicit	Universal authority, infallibility or neutrality of any one standpoint	Treating lived experience as uniquely biasing while professional expertise is presumed neutral, or treating any standpoint as infallible

## 3. Epistemic injustice, ignorance culture, exclusion culture and defensiveness

Epistemic injustice concerns wrongs done to people in their capacity as knowers [64]. In mental healthcare, stereotypes of irrationality, unreliability or manipulation can diminish credibility while professional interpretations receive presumptive authority [2,3,8,65]. Palafox-Harris analyses how psychiatric labels can activate perceptions of untrustworthiness [66]; Bueter examines exclusion from psychiatric classification [5]. These epistemic processes become materially consequential when interpretations influence records, treatment access, coercion or opportunities to contest decisions.

These dynamics are institutional as well as interpersonal. Newbigging and Ridley [4] show how institutional arrangements can negate epistemic agency under detention and unequal power. Landry [13] traces psychiatric survivor research partly to structures in which people with lived experience were positioned as subjects rather than decision-makers, while Logan and Karter [14] describe participation that can be tokenised without redistributing interpretive authority. Contemporary co-production work similarly argues that experiential knowledge must influence interpretation and strategic decision-making rather than function as representation alone [56]. A service can therefore invite lived experience participation while retaining professional control over what counts as a valid problem, outcome or explanation.

Ignorance culture and exclusion culture describe ways these structures can become self-maintaining. Downs [67] uses ignorance culture for the institutional handling of alarms through silence, minimisation, deflection or blame. Beale [68] describes exclusion culture as practices that make denying or withdrawing care appear clinically appropriate. Warrender's qualitative analysis of borderline personality disorder documents competing meanings through which people may be regarded as ‘not real mental illness,’ ‘only personality disorder’ or ‘too unstable for therapy,’ with consequences for treatment and sense of self [69]. These concepts direct attention to patterned processes: what is heard, what is recorded, what is coded as a symptom rather than a safety signal and what happens to people who leave because care feels unsafe [44,45].

Defensiveness names a related but distinct process: scrutiny of a practice or institution is redirected toward the credibility, pathology, emotionality or conduct of the person raising the concern. The focus is functional rather than psychodynamic. Defensiveness is not synonymous with countertransference, ordinary disagreement or bad faith; it becomes relevant when responses to challenge protect an established interpretation, treatment, diagnostic construct or institution from scrutiny [45,46,67,70].

Such responses can operate at clinical and organisational levels. Published accounts describe reports of harm being minimised or attributed to the person rather than prompting examination of care [44–47]. Lawson and Farquharson interviewed eight clients and seven staff selected for experience of negative effects from dialectical behaviour therapy. Their qualitative analysis identified pathologisation, retraumatisation and differing explanations of responsibility [71]. Accounts of service exclusion and discontinuation also question whether institutional responses adequately examine harm and poor fit [67,70]. The Royal College of Psychiatrists (RCPsych), in its position statement on adult survivors of non-recent child sexual abuse, acknowledges avoidable harm arising through routine practices and healthcare structures, including retraumatisation and inappropriate care [72]. Unintentional harm and institutional responsibility are therefore compatible.

Unequal interpretive authority has material consequences. A clinician's account can enter the medical record and shape diagnosis, risk assessment, coercion or access to care; a researcher's choices can determine which outcomes are foregrounded; and institutions can decide whether complaints become part of safety systems. Reflexivity must therefore extend to the structures that interpret patient testimony, not only to the people providing it. When a report of harm conflicts with an established model, a learning system should be capable of investigating both the report and the model, including revising the latter when evidence warrants it [4,32,70].

## 4. Diagnostic weaponisation and diagnostic recursivity

Diagnostic weaponisation denotes an unwarranted extension of diagnostic authority that discredits, excludes, coerces, punishes or controls. Its defining feature is not merely an adverse consequence but the use of a label beyond its evidentiary scope or as a substitute for an independently justified, proportionate decision. Diagnosis-informed risk management is not inherently weaponising; professional or legal authorisation, however, does not itself settle whether a particular use is justified. The process may be deliberate or structural. Faber et al. analyse racialised weaponisation in psychosis [73]; Smith et al. discuss political psychiatric abuse [74]; Harding describes weaponisation of personality disorder terminology [75]; and Cobbaert et al. analyse exclusionary and credibility-altering functions of borderline personality disorder discourse [36].

Diagnostic disparities warrant scrutiny of both classification and context. Garb reviews race and gender bias across diagnostic domains [76]. Shalaby et al. found that non-Hispanic Black individuals were less often diagnosed with attention-deficit/hyperactivity disorder and more often with conduct-related disorders than non-Hispanic White individuals [77]. Brown et al. identify inadequate contextual and demographic reporting in conduct disorder research and warn against diagnostic practices contributing to criminalisation [78]. Faber et al. situate contemporary psychosis disparities within historical pathologisation of Black civil-rights resistance [73], while Bhui discusses racialised stereotypes in risk assessment and coercive care [79]. These sources identify different mechanisms and evidentiary problems; a diagnostic disparity alone does not establish bias in every individual decision.

Borderline personality disorder provides a further example. Rodriguez-Seijas et al. found higher diagnosis rates among lesbian, gay and bisexual patients after adjustment for relevant clinical correlates [80]. Their later study compared transgender and gender diverse patients with cisgender heterosexual patients; the disparity persisted after adjustment for personality pathology specific to that diagnosis [81]. These observational findings raise concerns about diagnostic bias without excluding minority stress as a contributor to distress. Separately, Lam et al.’s experiment found that a suggested borderline personality disorder diagnosis adversely influenced clinicians’ judgements of uncomplicated panic disorder beyond equivalent behavioural information [82]. The issue is whether a label validly and equitably characterises distress and improves care, rather than whether people need support [36,83].

The consequences of classification extend beyond its initial assignment. Baker and Beazley's systematic review identifies negative clinician attitudes towards people diagnosed with borderline personality disorder [84]. Watts analyses testimonial quieting and smothering [85], and Palafox-Harris examines credibility deficits associated with psychiatric untrustworthiness stereotypes [66]. Warrender's qualitative discussion describes diagnostic overshadowing, conflicting meanings and exclusion from therapy [69]. These accounts support examining when diagnostic information is overextended into unwarranted judgements of credibility or deservingness of care. The analytic task is to trace how a label affects the evaluation of testimony and decisions about care.

Diagnostic recursivity concerns a self-confirming interpretation: a challenge to a diagnosis, treatment or service is taken as confirmation of the framework being challenged rather than evaluated on its merits. Emotional dysregulation, manipulation, splitting, resistance and lack of insight arise from different clinical traditions. Their relevance is a possible shared epistemic function, not an identical mechanism: each can, in particular circumstances, recast disagreement as evidence about the speaker. Aves describes concerns being redirected towards the author's personality and thinking [44]; Watts and Cobbaert et al. examine epistemic closure in borderline personality disorder discourse [36,85]. Palafox-Harris analyses self-reinforcing distrust [66], while Onstad considers restrictions on interpretive agency [43]. Recursivity is established by the circular handling of a challenge, not by the presence of a diagnostic label or clinical term alone.

## 5. Iatrogenic harm: Benefit is not safety

Effectiveness and safety require complementary assessment. An intervention can achieve an intended outcome and still cause harm; efficacy alone cannot establish safety [86,87]. Psychological-treatment harms remain inconsistently defined and assessed, limiting risk-benefit evaluation and informed consent [18–21,25–27]. Their heterogeneous definitions make prospective ascertainment and careful causal attribution more informative than an undifferentiated prevalence figure.

Safety depends on the person receiving an intervention and the conditions of its delivery. Aggregate findings may obscure harms concentrated in particular exposures or communities. Neurotype, culture, identity, trauma history and communication needs can affect treatment fit and what is experienced as harmful [22,28,30,41]. The RCPsych statement likewise identifies risks when trauma and context are inadequately recognised [72]. Population evidence should inform collaborative, person-specific monitoring and adaptation rather than replace them.

Safety assessment also requires a sufficiently complete evidence record. Studies of selective dissemination demonstrate why published benefits may be misleading [50,51,62]; where harms are not adequately sought, neither a favourable efficacy estimate nor silence about adverse outcomes resolves uncertainty about safety [18–21].

Benefit and harm can coexist within one episode of care. Sandsten et al.’s qualitative study of 18 women describes general psychiatric care for anorexia nervosa as life-saving yet also unsupportive, coercive or enduringly harmful [88]. Spadaccini and Schweizer's lived experience narrative describes medical stabilisation alongside trauma and prolonged healthcare avoidance following gender identity conversion practices in eating disorder treatment [89]. Alford et al. interviewed nine autistic people about adolescent treatment and nine parents, identifying misattunement, trauma, mistrust and recovery setbacks [28]. These accounts identify outcomes that symptom improvement alone cannot capture.

Comparable issues arise beyond eating disorders. Shipwright and Murphy interviewed seven people reporting long-term adverse effects after electroconvulsive therapy, including memory changes, losses of identity and dismissive professional responses [90]. Two Read et al. papers report outcomes and recalled pretreatment information from the same convenience survey of 858 recipients and 286 relatives or friends [91,92]. The shared convenience sample supports descriptive analysis rather than independent replication or population prevalence estimates. These accounts identify questions about long-term effects, information provision and follow-up that systematic safety research needs to address.

Antidepressant withdrawal illustrates how recognition shapes safety knowledge. Guy et al.’s analysis of patient accounts identifies dismissal, misdiagnosis and inadequate tapering support [93]. Horowitz et al. found withdrawal symptoms in 79% of 310 respondents who had attempted discontinuation; the 18% response rate limits population inference, although longer use was associated with greater severity and duration [94]. Tarchi et al.’s systematic review documents persistent sexual difficulties after selective serotonin reuptake inhibitor discontinuation, but could establish neither reliable prevalence nor a cause-effect relationship [95]. Healy and Mangin identify under-recognition and reporting barriers that impede quantification [96]. Uncertainty calls for better ascertainment, not dismissal of reported experiences.

Safety information also depends on who remains visible. Aves's narrative describes service avoidance following harm [44]. A plausible consequence is that monitoring restricted to continuing service users misses experiences that explain disengagement. Complaint counts are likewise not prevalence estimates: fewer reports may reflect safer care, but also diminished trust or barriers to reporting. Services need voluntary, accessible ways to hear from people who leave without interpreting disengagement itself as evidence that their accounts lack credibility.

Informed consent depends on information about benefits, harms, alternatives and uncertainties. Allison et al. argue that inadequate psychotherapy adverse-effect evidence constrains consent [25]; patient accounts identify information gaps concerning antidepressant withdrawal [93] and electroconvulsive therapy [92]. A conscientious clinical conversation cannot compensate fully for an incomplete safety evidence base. Both the evidence and the communication of its limitations require scrutiny.

## 6. Coercion, misconduct and the institutional learning test

Coercion and sexual misconduct differ from diagnostic bias and treatment adverse effects, but also test accountability under asymmetric power. Frueh et al. document patient reports of traumatic experiences in psychiatric settings [97]. In South Australia, Brayley et al. found that 60% of involuntary admission forms did not document all legal criteria [98]; this is evidence of deficient recording, not proof that 60% of admissions lacked legal grounds. Lived experience synthesis identifies psychological and human rights consequences of seclusion and restraint [99]. Bhui examines racialised risk assessment [79], and the RCPsych statement acknowledges retraumatisation associated with restrictive practices [72]. Authorisation therefore does not remove the obligation to scrutinise necessity, proportionality, alternatives and experienced harms.

Professional misconduct presents another warning signal. Dehlendorf and Wolfe describe historical US disciplinary actions for sexual offences [100]. Alam et al. compare Canadian psychiatrists’ disciplinary patterns with those of other physicians [101]. Bismark et al. analyse Australian notifications alleging sexual misconduct and identify elevated notification rates in psychiatry [102]. Notifications are not equivalent to substantiated misconduct, and different regulatory denominators cannot yield a pooled prevalence. These studies instead warrant scrutiny of prevention, reporting, investigation and institutional responses.

Kröger et al.’s stakeholder research identifies roles for rules, dialogue and organisational culture in preventing sexual boundary violations [103]. Such recommendations should be distinguished from evidence that particular safeguards reduce recurrence. Institutional learning requires transparent accounts of implementation, evaluation and outcomes, alongside education, supervision and professional reflection.

Across these domains, we propose institutional non-learning as a shared systems vulnerability. Interpretive authority can influence credibility, recognition of harm, documentation and access to corrective action. Fig 1 presents this conceptual cycle and proposed points of interruption.

**Fig 1:**
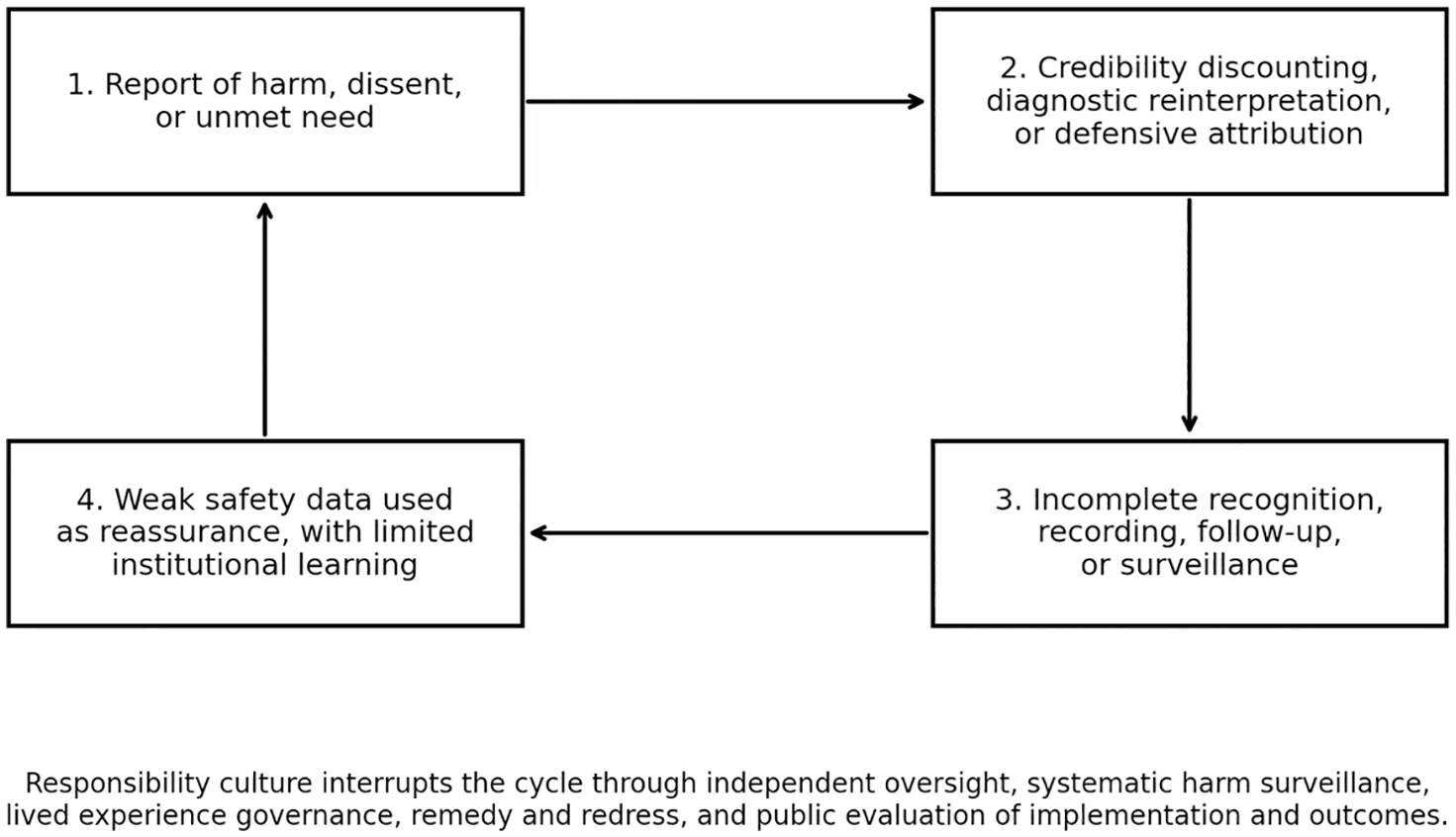
Conceptual cycle of institutional non-learning. Different harm domains may enter the cycle at different points. The proposed responsibility culture aims to interrupt it through independent scrutiny, active harm surveillance, lived experience governance, remedy and redress, and public evaluation of implementation and outcomes.

## 7. Responsibility culture: What counts as adequate institutional learning?

A responsibility culture turns epistemic humility and reciprocal reflexivity into institutional practice. It evaluates systems by their capacity to detect, investigate, repair and reduce avoidable harm, distinguish necessary treatment burden from preventable iatrogenesis, respond fairly to contested accounts and demonstrate whether safety improves over time. Accountability is therefore assessed through functions and outcomes that can be observed and evaluated.

Four linked standards follow. First, serious or recurrent concerns require scrutiny sufficiently independent to challenge the institution. Second, safety surveillance must actively solicit clearly defined harms. Third, relevant lived experience expertise needs remunerated decision-making authority over harm definitions, outcomes, priorities and evaluation. Survivor scholarship warns against participation without redistributed authority [12–14]; workforce and co-production frameworks develop practical routes to recognition and influence [53,55,56]. Fourth, institutions need mechanisms for remedy, redress and public reporting of implementation. Elvidge et al.’s Indigenous-led framework offers a related, specifically Aboriginal and Torres Strait Islander account of governance, cultural safety, data sovereignty and organisational accountability [104].

Independence requires epistemic as well as organisational distance. Reviewers outside a case may still share the assumptions or allegiances under examination [32,34–36]. Cross-disciplinary, cross-sector and lived experience governance can widen the interpretive frame [4,56]. This is a design requirement for meaningful scrutiny, not an assurance that any particular panel is free from bias. Indigenous governance and data sovereignty must also be respected rather than subsumed into generic participation [104].

Monitoring should combine patient-reported experiences, clinical deterioration, adverse events and treatment exposure, with proportionate follow-up [24,105]. Reasons for discontinuation and service avoidance are also relevant [44,70]. Contact after disengagement must be voluntary and accessible. Elvidge et al. specifically locate authority to judge cultural safety with Aboriginal and Torres Strait Islander people receiving care, rather than institutions relying on training or policy counts [104]. This complements, rather than replaces, clinical safety assessment. Indicators should be stratified by relevant groups and settings where ethical and adequately supported by the data; unmeasured or sparse subgroups require explicit uncertainty.

Research transparency is part of safety surveillance. Registration, accessible protocols, complete outcome reporting and publication regardless of results support scrutiny of what later enters reviews and guidelines [49–52,62]. Longstanding recognition of reporting bias is not evidence of improvement. Progress requires longitudinal assessment of publication completeness and protocol-publication concordance.

Policies, training and commitments are inputs, not evidence that safety has improved. A credible accountability cycle identifies a problem, assigns responsibility, specifies actions and timescales, evaluates outcomes and revises unsuccessful interventions. It also corrects records or decisions and provides remedy where warranted. Elvidge et al. propose related requirements for organisational responses and community-governed monitoring [104]. Table 2 sets out operational standards against which implementation of the proposed responsibility culture can be evaluated.

**Table 2:** Proposed operational standards for a responsibility culture.

Standard	Who must act	Required practice	Observable evidence of implementation
1. Independent scrutiny	Regulators, independent assessors, statutory or cross-jurisdictional bodies, lived experience governance	Review recurrent or serious harm with sufficient epistemic distance; conduct cross-case analysis; publish de-identified findings	Independent review reports; cross-case analyses; named prevention actions; evidence that recommendations were implemented
2. Active safety surveillance	Services, researchers, funders, journals and safety bodies	Predefine and actively solicit harms; include communities exposed to specific risks; analyse by population, setting and treatment exposure; use intersectional analyses where feasible; offer voluntary follow-up after disengagement; preregister outcomes and report results regardless of direction	Prospective safety protocols; measures with appropriate validity evidence; subgroup and intersectional analyses; documented inclusion of relevant communities; adverse-event reporting; longitudinal follow-up; reasons for dropout and service avoidance; trial registration; protocol-publication concordance; publication of null and unfavourable findings
3. Lived experience authority	Governance boards, research teams, professional bodies and services	Recognise lived experience as domain-specific expertise and provide remunerated decision-making roles in defining harm, setting priorities, interpreting data and evaluating reform	Paid governance roles; documented decision authority; explicit reporting of involvement; evidence that lived experience expertise changed measures, policy or practice
4. Remedy, redress and public learning	Services, professional bodies, regulators and funders	Correct records or diagnoses where warranted; restore access; provide apology or redress where appropriate; publish implementation milestones and outcomes	Record corrections; remedies; implementation dashboards; outcome trends; explanations for delayed or unsuccessful reform

## 8. Conclusion

Psychiatric expertise has value, but professional status does not confer epistemic neutrality. Its institutional authority makes reciprocal scrutiny particularly important: interpretations can enter records, shape treatment access, justify coercion and determine whose concerns are investigated [2,4,32]. Lived experience and professional expertise offer different, sometimes overlapping forms of access to care. Neither should be presumed infallible, and neither should be excluded from evaluating the other's claims.

Epistemic reciprocity requires matching evidence to questions, distinguishing benefit from safety, justifying extrapolation and making interpretive assumptions visible. It also requires institutions to ask whose experiences are missing and whether testimony can alter the definitions, measures and practices through which harm is understood [9,32,36,56]. A responsibility culture makes that commitment evaluable through independent scrutiny, active surveillance, lived experience authority, remedy and evidence of implementation and outcomes.

Trust should rest on transparent self-correction rather than assurances of good intent. The practical test is whether institutions can recognise when their own practices are implicated, respond to those affected and demonstrate what changed.
